# Supplementary material for: Exposure to low levels of heavy metals and chronic kidney disease in the US population: A cross sectional study
Source: PLoS One. 2024 Apr 16;19(4):e0288190. doi: 10.1371/journal.pone.0288190 (PMC11020388; doi:10.1371/journal.pone.0288190)
Supplement: Attachment — Submitted filename: Response to reviewers 02-06.docx [file pone.0288190.s005.docx]

Manuscript Number: PONE-D-23-18872

Title: EXPOSURE TO LOW LEVELS OF HEAVY METALS AND CHRONIC KIDNEY DISEASE IN THE US POPULATION: A CROSS SECTIONAL STUDY

Dear Editor,

Thank you and the reviewers for the constructive suggestions and reviews. We have made changes in the revised manuscript to reflect the editor’s and reviewers’ comments.

**Reviewer #2:**

1. **What are the possible explanations for the findings with cotinine?**

**Thank you for the suggestion. The reason behind the inverse relationship between blood cotinine level and CKD is unclear at this moment. Different studies have found contrasting relationships between them. Further studies would need to be done to explore the relationship.**

2. **Provide the reference for the classification of CKD. (KDIGO guidelines/KDOQI?)**

**Thank you for the suggestion. The CKD classification has been referenced with k/DOQI.**
